# Effect of Processing on Fish Protein Antigenicity and Allergenicity

**DOI:** 10.3390/foods10050969

**Published:** 2021-04-28

**Authors:** Xingyi Jiang, Qinchun Rao

**Affiliations:** Department of Nutrition, Food and Exercise Sciences, Florida State University, Tallahassee, FL 32306, USA; xj15@my.fsu.edu

**Keywords:** fish allergy, parvalbumin, antigenicity, allergenicity, immunogenicity, linear epitope, conformational epitope, processing, immunoglobulin G (IgG), immunoglobulin E (IgE), calcium-binding protein

## Abstract

Fish allergy is a life-long food allergy whose prevalence is affected by many demographic factors. Currently, there is no cure for fish allergy, which can only be managed by strict avoidance of fish in the diet. According to the WHO/IUIS Allergen Nomenclature Sub-Committee, 12 fish proteins are recognized as allergens. Different processing (thermal and non-thermal) techniques are applied to fish and fishery products to reduce microorganisms, extend shelf life, and alter organoleptic/nutritional properties. In this concise review, the development of a consistent terminology for studying food protein immunogenicity, antigenicity, and allergenicity is proposed. It also summarizes that food processing may lead to a decrease, no change, or even increase in fish antigenicity and allergenicity due to the change of protein solubility, protein denaturation, and the modification of linear or conformational epitopes. Recent studies investigated the effect of processing on fish antigenicity/allergenicity and were mainly conducted on commonly consumed fish species and major fish allergens using in vitro methods. Future research areas such as novel fish species/allergens and ex vivo/in vivo evaluation methods would convey a comprehensive view of the relationship between processing and fish allergy.

## 1. Introduction

Food allergy is an adverse immune response to food, which can be classified into immunoglobulin (Ig) E-mediated and non-IgE mediated [1]. In IgE-mediated food allergy, IgEs bind to the food allergens, leading to the granulation of immune effector cells, releasing histamine and other inflammatory mediators [2]. Food allergy can be diagnosed using clinical disorders, physical examination such as serum total/specific IgE measurement, a skin prick test (SPT), and oral food challenge (OFC) [3,4]. It is reported that food allergy affects around 2.5% of the worldwide population, and its prevalence is increasing over time [5]. In the U.S., at least 10% of adults and 8% of children [6] have a food allergy. Some prevention strategies such as ingestion of potential allergens during pregnancy [7], consumption of prebiotics/probiotics/symbiotics/bacterial lysates [8], and vitamin D supplementation [9] are suggested. However, there is no treatment for food allergy, except for peanut allergy, which can be alleviated by oral immunotherapy with PALFORZIA (Aimmune Therapeutics, Inc., Brisbane, CA, USA) [10].

According to the World Allergy Organization (WAO), peanuts, tree nuts, finned fish, shellfish, milk, egg, wheat, soy, and sesame are the foods causing the most significant allergic reactions [11]. Among them, the estimated prevalence of finned fish allergy worldwide and in the U.S. is 0.3% [12] and 0.3–0.9% [13,14], respectively. Fish allergy usually persists throughout life, and its prevalence is unlikely to decrease or become stable [15]. The symptoms of fish allergy vary from mild to severe and may even lead to death. As an immunoglobulin (Ig) E-mediated food allergy, fish allergy is an adverse response when IgE binds to the ingested fish allergens [1]. According to the WHO/IUIS Allergen Nomenclature Sub-Committee [16], many fish proteins, including beta parvalbumin, beta enolase, aldolase A, tropomyosin, collagen alpha, creatine kinase, triosephosphate isomerase, pyruvate kinase, beta-prime-component of vitellogenin, PKM-like L-lactate dehydrogenase, glucose 6-phosphate isomerase, and glyceraldehyde3-phosphate dehydrogenase, have been recognized as food allergens. It should be noted that due to the variety of fish species from different regions and under-investigated fish species, more fish allergens from different species are continuously being submitted to the WHO/IUIS [17,18]. Among the currently reported fish allergens, parvalbumin IgE epitopes have been reported from many species such as cod [19,20,21], Pacific mackerel [22], Atlantic salmon [23], and Asian seabass [24]. IgE epitopes from other fish allergens are seldom reported. Many studies pointed out the clinical importance of characterizing other important fish allergens [18,25,26]. Fish and fishery products undergo different food processing techniques to inactivate pathogenic microorganisms, destroy toxins, and improve the taste. It is well known that protein structure and functional properties can be changed during different processing methods. For example, treatment such as drying, heating, and smoking leads to protein denaturation and protein solubility impairment [27]. Fish protein hydrolysates from chemical or enzymatic hydrolysis contain shorter peptides or amino acids that are easily absorbed [28]. Food processing techniques could lead to a decrease, no change, or even increase in fish antigenicity and allergenicity. Generally, both in vitro and in vivo methods are used to evaluate the effect of food processing on the properties of fish proteins. For in vitro methods, gel electrophoresis is used to study the soluble proteins’ conformation, stability, and interaction. Enzyme-linked immunosorbent assays (ELISAs) and immunoblots are applied to study fish proteins’ antigenicity and allergenicity. For example, de Jongh et al. [29] used gel electrophoresis to study parvalbumin glycation and its digestion stability. Kubota et al. [30] applied ELISA and Western blotting to demonstrate the weakened thermostability of Pacific mackerel parvalbumin. For in vivo methods, SPT [31] and OFC [32] were used to illustrate patients’ immune responses to fish collagen.

In this concise review, three aspects of fish allergy (terminology of immunogenicity, antigenicity, and allergenicity; the epitopes of fish allergens; and effect of food processing on fish antigenicity/allergenicity) are elaborated.

## 2. Immunogenicity, Antigenicity, and Allergenicity

When studying the effect of food processing on allergens, terms such as immunogenicity, antigenicity, and allergenicity are often used interchangeably. Immunogenicity is the ability of a substance to induce a cellular or humoral immune response under a given set of conditions [33]. Immunogenicity is described in terms of the following three aspects: (1) the ability to defend the immune system; (2) the ability to keep the immune system steady; (3) the ability to kill or to remove abnormally mutated cells [34]. Antigenicity is defined as “the capacity to combine specifically with antibodies or T-cell receptor/major histocompatibility complex (MHC)” [33]. It is the ability to induce an immunological response [34]. From our perspective, protein antigenicity can be described from in vitro experiments such as Western blots and enzyme-linked immunosorbent assays (ELISAs). Both immunoglobulin (Ig) G or IgE can be used for in vitro antigenicity studies. For IgG, either a monoclonal antibody (mAb) that binds to the same epitope of the protein or a polyclonal antibody (pAb) that binds to multiple epitopes of the same protein is applied. For example, frying increased anti-shrimp tropomyosin mAb immunoreactivity [35], while glycated parvalbumin showed a decrease in antigenicity using pAb [36]. As for IgE, pooled human sera [37] or individual serum containing IgE [38] are also reported. Protein immunogenicity can be characterized in vitro by analyzing its ability to produce T and B cell responses during the allergic sensitization phase [39]. For example, Ilchmann et al. [40] reported an activation and proliferation of T cells after glycation of ovalbumin. Cooking crustacean shellfish did not change T cell proliferative or cytokine reactivity in allergic patients’ peripheral blood mononuclear cells [41].

According to the WAO, allergy is a hypersensitivity reaction initiated by a specific immunologic mechanism [1]. Food allergy is an adverse immunologic response to food proteins [42]. To the best of our knowledge, no official definition was given for food allergenicity. The authors specify their individual descriptions in each publication. For example, according to the European Food Safety Authority (EFSA), allergenicity is “the ability of an antigen to induce an abnormal immune response, which is an overreaction and different from a normal immune response in that it does not result in a protective/prophylaxis effect but instead causes physiological function disorder or tissue damage” [34]. Deifl and Bohle [43] defined it as “the property of being able to induce a type 2 T helper (Th2) response and subsequent production of allergen-specific IgE antibodies.” Allergenicity could also be considered as “specifically bind IgE using sera from individuals with clear allergies to the source of the gene/protein and further that the protein causes basophil activation or histamine release, skin test reactivity or challenge test reactivity using subjects allergic to the source” [44] or “the ability of an antigen to induce an abnormal immune response, which is an overreaction and different from a normal immune response in that it does not result in a protective/prophylaxis effect but instead causes physiological function disorder or tissue damage” [45]. The term “food protein allergenicity” should be used carefully because it emphasizes the development of adverse reactions in skin, respiratory, digestive, and circulatory organs. Food protein allergenicity should be described using in vitro and in vivo methods according to the WHO/IUIS allergen submission requirement [46]. First, in vitro IgE tests such as ImmunoCAP, ELISA, and immunoblotting could show what the body is reacting to [47]. A basophil activation test is a replacement to measure the markers on the surface of basophils following stimulation with the allergen [48]. Second, a skin prick test or an in vivo allergen challenge test could also be applied. As an example, Faeste et al. [49] applied specific serum IgE determination, a skin prick test, and an open food challenge to illustrate the allergenicity of fenugreek proteins. Overall, to avoid confusion and improve multidisciplinary communication, accurate and consistent terminology and the recommended methods for studying food protein immunogenicity, antigenicity, and allergenicity should be developed.

## 3. Fish Allergy and Allergens

### 3.1. Fish Allergy Prevalence

Fish allergy usually has the following characteristics. First, fish allergy can happen due to ingestion, skin contact, or even inhalation exposure in the occupational environment [50] and is typically a life-long illness [51]. In this review, only ingested fish allergy will be discussed. The prevalence of fish allergy is summarized in Table 1. Fish allergy prevalence is affected by demographic factors such as region, age, gender, and ethnicity. Generally, Asians, adults, and females have a higher chance of developing fish allergies than those in Western countries, children, and males, respectively. Second, cross-reactivity in fish allergy is more common compared to other food allergies, such as wheat and egg. A person with fish allergy has a 50% possibility of being allergic to more than one fish [52]. It is suggested that patients who have fish allergies should avoid all types of fish in their diet [53]. In addition, fish allergic patients were also reported to be allergic to shellfish [54], chicken [55], and frogs [56] due to protein (such as parvalbumin and collagen) cross-reactivity. Third, fish allergy is one of the leading causes of food anaphylaxis [57]. It was found that fish accounted for 9% of deaths from anaphylaxis [58]. Pitsio et al. [59] first reported two cases of anaphylaxis during the SPT using commercial fish extracts.

Most of the fish allergy prevalence studies used a self-reported questionnaire-based method or telephone survey. Other methods such as an SPT, serum IgE measurement, and the gold standard DBPCFC criterion are seldom reported (Table 1). There are some adverse reactions such as scombroid fish poisoning [60] and fish parasite *Anisakis simplex* allergy [61] that are similar to the symptoms of fish allergy, which may lead to deviation of the prevalence.

**Table 1:** Prevalence of fish allergy.

Target	Method	Prevalence (%)	Comment	Reference
5529 households	Telephone survey	0.4	Adults have a higher prevalence than children Females have a higher prevalence than males	[62]
574 adults (>18 yr)	Telephone survey	0.8		[63]
38,480 U.S. children	Telephone survey	0.5		[6]
20,686 U.S. participants	Self-report survey	0.45	Adults have a higher prevalence than children	[14]
7218 U.S. households (>18 yr)	Telephone survey	0.9	Finfish allergy is likely to be developed in adulthood	[13]
11,434 children in the Philippines (14–16 yr)	Questionnaire survey	2.9	Females have a higher prevalence than males	[64]
6498 children in Singapore (14–16 yr)	Questionnaire survey	0.26	Females have a higher prevalence than males	
2304 children in Bangkok (14–16 yr)	Questionnaire survey	0.29	Females have a higher prevalence than males	
9667 individuals in Canada	Telephone survey	0.61	Cod and salmon are most reported allergenic species	[65]
3500 children in Turkey (6–9 yr)	Questionnaire	3.5		[66]
	Skin prick test	5.6		
	DBPCFC ^†^	4.5	Only one child was positive in the DBPCFC	
9184 children in low-income clinic (0–21 yr)	Medical records	0.4	Fish is the second species group that easily causes anaphylaxis	[67]
30,018 individuals in Taiwan, China	Questionnaire	19	Mostly occurred in children between 4-18 yr	[68]
430 children in Poland with asthma	DBPCFC	0.3	The prevalence of fish allergy in Poland was relatively low	[69]
22 Chinese patients with fish allergy	DBPCFC	71.4	17.8% of patients were allergic to both carp and salmon	[70]

DBPCFC ^†^: Double-blind placebo-controlled food challenge.

### 3.2. Fish Allergens

According to the WHO/IUIS Allergen Nomenclature Sub-Committee [16], fish proteins including beta parvalbumin, beta enolase, aldolase A, tropomyosin, collagen alpha, creatine kinase, triosephosphate isomerase, pyruvate kinase, beta-prime-component of vitellogenin, PKM-like L-lactate dehydrogenase, glucose 6-phosphate isomerase, and glyceraldehyde 3-phosphate dehydrogenase have been recognized as food allergens. Many review articles have been published on the structure and physicochemical characterization of fish allergens [51,71,72,73]. The reported fish allergen IgE-binding epitopes are summarized in Table 2. The epitopes are usually mapped using synthetic peptides, while this method cannot generate conformational epitopes. It is noted that the IgE-binding epitopes for fish allergens other than parvalbumin are seldom reported. For fish parvalbumin, both linear and conformational IgE epitopes from different species have been recognized (Table 2). Its IgE epitopes were mainly found in EF-hand motifs which are capable of binding with calcium and magnesium ions [74]. Despite the relatively low amino acid similarity among fish and other vertebrate animals, the high resemblance (~90%) in CD and EF domains, i.e., metal-binding sites, is noticed. This is probably why fish allergic patients can develop symptoms to more than one fish species or even other vertebrate animals, such as frogs and chicken.

**Figure 1:**
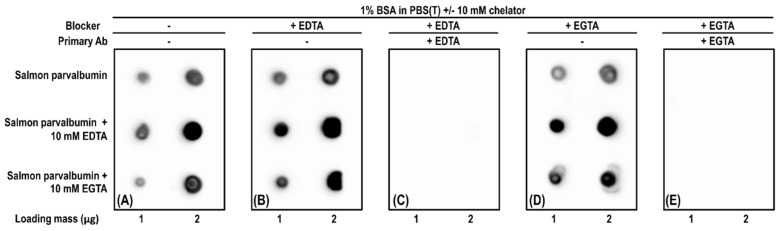
Effect of chelator on antibody-antigen interaction using dot blot. (**A**,**E**) Dot blot using mAb PARV19 (monoclonal anti-parvalbumin antibody, Sigma-Aldrich, P3088). (**B**,**C**) and (**D**,**E**) membranes were blocked using 1% (g/mL) BSA (bovine serum albumin) in PBS (10 mM phosphate-buffered saline, pH 7.2) containing 10 mM EDTA or 10 mM EGTA, respectively. (**C**,**E**) membranes were incubated with PARV19 diluted in 1% BSA in PBST (0.05% (mL/mL) Tween 20 in PBS) containing 10 mM EDTA or 10 mM EGTA, respectively.

Calcium ions play an important role in parvalbumin IgE-binding properties due to its ability to keep parvalbumin’s conformation. Many studies showed a decrease in the IgE-binding ability after calcium depletion using ELISA and Western blots (Table 3). To study the effect of calcium on parvalbumin–antibody interactions, chelators such as ethylenediaminetetraacetic acid (EDTA) or ethylene glycol-bis(β-aminoethyl ether)-*N*,*N*,*N*’,*N*’-tetraacetic acid (EGTA) are often added. However, there are two concerns. First, it is crucial to verify if the calcium ion has been removed completely. It is commonly accepted that EGTA has a higher affinity to calcium ion than EDTA. Methods such as fluorescence spectrum determination using Quin 2 [30] or conformation analysis using circular dichroism [77] should be applied. Second, chelators are not recommended to coexist with both the target protein and antibody due to their interference with antibody–antigen interactions. For example, we used a commercial mouse anti-parvalbumin mAb (PARV19, Millipore Sigma, P3088), which has a calcium-dependent epitope [78], to illustrate as follows. From the dot blot (Figure 1), three major findings were obtained. First, salmon parvalbumin immunoreactivity increased when 10 mM EDTA or 10 mM EGTA was incubated with purified salmon parvalbumin (Figure 1A), which matched our previous findings [23]. Additionally, Gajewski and Hsieh [79] reported an increase in mAb PARV19 immunoreactivity with calcium-depleted fish protein extracts. Second, when chelators were only added to the blocker, immunoreactive parvalbumin was still visible, and its dot intensity was not different from the one blocked without chelators (Figure 1A,B,D). It is also noticed that neither EDTA nor EGTA could affect the immunoreactivity. Third, when EDTA and EGTA were also added to the primary antibody buffer, which contained mAb PARV19, the parvalbumin dots disappeared (Figure 1C,E). From this research, it was found that chelators such as EDTA and EGTA not only chelate calcium ions but also may affect the antibody–target interaction. Any false positive/negative detection results should be carefully evaluated when chelators are added in the presence of antibodies.

## 4. Effect of Processing on Fish Allergens

Fish are more perishable than other high-protein animal meat due to the high concentration of nonprotein nitrogenous compounds present [83]. Food processing is directed to (1) avoid spoilage and decrease foodborne diseases; (2) increase food tastes and nutritional values; (3) improve transportation stability; and (4) produce convenient food [84]. Both thermal and non-thermal processing techniques are applied to fish products to increase shelf life and enhance sensory properties (Table 4). After processing, fish protein structure [85], stability [86], and antigenicity/allergenicity [87] can be altered.

Table 5 summarizes the effect of food processing on fish antigenicity/allergenicity. Overall, three major conclusions can be driven. First, fish antigenicity/allergenicity is affected by a number of factors (matrix, detection method, antibody). The antigenicity/allergenicity of the same protein exhibits differences in different matrices. Griesmeier et al. [88] reported IgE binding to heated (100 °C/10 min) whiff proteins even after in vitro pepsin digestion for 120 min while the IgE binding to heated whiff parvalbumin monomer disappeared after a 5 s digestion. Keshavarz et al. [23] noticed parvalbumin was almost undetectable in heated (100 °C/8 min) salmon protein extracts, while purified salmon parvalbumin was thermostable and soluble after the same heat treatment.

Indirect ELISAs and Western blots are the methods that are commonly used to study the effect of processing on fish allergens. These methods mainly investigated the binding between antibodies and processed proteins that may lead to a modified capacity to elicit an allergic reaction [89]. The reliability of the results is dependent on the extractability of fish allergens and the selection of antibodies. First, both ELISAs and Western blots rely on the extractable fish proteins, whose amount is affected by the extraction condition. Generally, the processed fish proteins are extracted using water or phosphate-buffered saline (PBS), which may not represent the total amount of allergens. Like other animal muscle proteins, fish proteins are classified as myofibrillar, sarcoplasmic, and stromal proteins, and the specific composition is species dependent [90]. Different extraction strategies should be conducted against different proteins’ properties to ensure their extractability. It is reported that a larger amount of IgE-reactive bands was observed from oyster when it was extracted using high-salt buffers or high-pH buffers [91]. The addition of 5 mM EDTA in the extraction solution increased salmon parvalbumin extractability significantly [23]. Water non-extractable parvalbumin from heated (100 °C/8 min) salmon was further extractable by adding a surfactant (SDS) and reducing agent (β-mercaptoethanol) [23]. Ma et al. [92] compared the parvalbumin extractability using 12 different buffers and noted that the reducing agent (dithiothreitol) enhanced extraction efficacy and led to higher stability and functionality of the protein extracts. Meanwhile, the antibody used affects the results. When IgGs are used as the detection antibody, this usually involves immunoreactivity changes of one single protein. When human IgEs are used, this reflects the total antigenicity/allergenicity. During the evaluation of the same product, IgGs and IgEs can lead to the same or different detection results. For example, decreased IgG and IgE immunoreactivities were observed after glycation parvalbumin with maltose [36] while a reduction in IgG binding and an increase in IgE binding were observed in heated (100 °C/10 min) sardine parvalbumin [93]. Despite the wide applications of immunoblots and ELISAs, few reports that focus on the ability of fish proteins to induce allergic sensitization have been published. In vitro experiments such as histamine release tests, mediator release tests, T cell polarization assays, cytokine production and proliferation [94], and in vivo tests such as skin prick tests and oral food challenges are all recommended.

**Table 4:** Summary of food processing techniques on fish products from selected publications.

Food Processing Technique	Property	Impact on Fishery Products	Reference
Cooking	Different strategies such as boiling, steaming, microwaving, baking, roasting, frying, and grilling are applied	Improve taste and flavor; affect texture and nutrition value; induce protein denaturation	[27]
Canning	Add food in jars and process in a pressure canner	Extend shelf life; induce flavor, texture, and nutrition loss	[27]
Hot smoking	Apply the smoke from burning materials such as wood at a temperature around 70–80 °C	Reduce moisture and microorganisms; impart desirable flavor	[95]
Drying	Remove water or other solvents by evaporation	Reduce moisture and microorganisms; induce protein denaturation; alter fish texture and color	[96]
Fermenting	Apply microorganisms to convert carbohydrates into different products	Extend shelf life; impart organoleptic and nutritional characteristics	[97]
Salting	Apply dry edible salt	Reduce moisture and microorganisms; induce lipid and protein degradation; alter fish texture and color	[98]
Cold smoking	Smoking of the product up to 33 °C	Less efficient in microbial reduction; alter texture, color, and flavor	[95]
High-pressure processing	Apply pressure between 200–800 MPa at a mild temperature of 5 to 35 °C	Inactivate microorganisms; induce protein denaturation; increase lipid oxidation; decrease water-holding capacity	[99,100,101]
Ultrasound	Apply an ultrasound frequency from 20 kHz to 10 MHz	Reduce microbials; affect color	[102,103]
Pulsed light	Apply short duration, high-peak power pulsed light of wide spectra (100–1100 nm)	Reduce microbials; affect color and texture; reduce lipid oxidation	[104,105]
Pulsed electric fields	Induce electroporation phenomena between two electrodes, leading to a non-invasive tissue structure modification	Improve water-holding capacity; tenderize texture; extraction of fishery by-products	[106]
Cold plasma	Apply energetic, reactive gases such as argon, helium	Reduce microbials; alter moisture content and lipid oxidation	[107,108]
Ozone	Works as a powerful oxidant and does not leave residues in foods	Reduce microbials; extend shelf life	[109]

**Table 5:** Effect of processing on fish protein solubility, antigenicity, and allergenicity.

Fish Matrix	Processing Method	Method	Antibody	Major Results	Explanation	Reference
Pacific mackerel protein extracts	60, 80, 100, 120, 140 °C for 5, 10, 15, 20, 25, 30 min	Western blot	mAb PARV-19	Parvalbumin band decreased as a function of heating temperature and time	The reduction was caused by heat-induced conformational change due to the release of calcium	[30]
		Indirect non-competitive ELISA	Human IgE	IgE reactivity decreased as a function of heating temperature and time; a complete loss of IgE reactivity at 140 °C		
Hilsa, pomfret, bhetki, and mackerel	90 °C for 10 min	Indirect non-competitive ELISA	Human IgE	A decrease in IgE reactivity was observed in pomfret, hilsa, and mackerel while an increase in IgE reactivity was seen in bhetki	Boiling removed many polypeptide bands	[110]
		Skin prick test		Patients exhibited different reactions to boiled fish		
	Fry with mustard oil for 5 min	Indirect non-competitive ELISA	Human IgE	A decrease in IgE reactivity was observed in pomfret, hilsa, and mackerel while an increase in IgE reactivity was seen in bhetki	Frying removed many polypeptide bands and caused protein denaturation to form high molecular weight proteins	
		Skin prick test		Patients exhibited different reactions to boiled fish		
Snapper, silver bream, yellowtail kingfish, barramundi, bluefin tuna, slimy mackerel, orange roughy, tiger flathead, Atlantic salmon, rainbow trout, carp, pilchard, rock ling, Atlantic cod,	95 °C for 15 min	Western blot	mAb PARV-19	Parvalbumin from heated fish was still immunodetectable. Especially for yellowfin tuna, a stronger and more intense parvalbumin band was observed compared to unheated extracts	Heat processing affected antibody–antigen interaction differently for each species	[111]
gummy shark, sparsely spotted stingaree, blacktip shark, and elephant shark	95 °C for 15 min	Western blot	mAb PARV-19	Except for elephant shark, immunoreactive parvalbumin was not visible		
Purified cod parvalbumin	80 °C for 30 min	Indirect non-competitive ELISA	Human IgE	IgE binding was not affected	Heat-induced secondary structure and calcium-binding ability changes were not enough to reduce antigenicity	[112]
	80 °C, 300 MPa for 30 min			IgE binding was not affected		
Bhetki and mackerel fish extracts	90 °C, 10 min then pepsin digested	Indirect non-competitive ELISA		No significant difference in IgE level was observed		[113]
		Western blot		Additional immunoreactive protein bands were observed	Thermal processing generated new allergenic epitopes that were pepsin stable	
	Fry in mustard oil for 5 min then pepsin digested	Western blot		Increased IgE binding proteins were observed	Structural changes may offer some protection from enzymatic digestion	
Surimi	100 °C for 10, 15, and 20 min	Indirect non-competitive ELISA and indirect competitive ELISA	Anti-fish tropomyosin mAb	IgG binding decreased after 10 min and remained constant for 15 and 20 min	High temperature and long processing time decreased extractable protein concentration, destroyed epitopes, and affected antibody–antigen interaction	[114]
	Steam at 100 °C for 10, 15, and 20 min			IgG binding decreased after 10 min and remained constant for 15 and 20 min		
	Bake at 149 °C for 10, 20, and 30 min			IgG binding decreased as a function of baking temperature		
	Microwave on high power for 0.5, 1, and 1.5 min			IgG binding decreased as a function of microwaving temperature		
	Fry in canola oil for 0.5, 1, and 1.5 min			IgG binding decreased after 10 min and remained constant for 15 and 20 min		
Purified cod parvalbumin	Glycation with D-glucose (60 °C for 5 h) and in vitro digestion	SDS-PAGE		All parvalbumin was digested after 30 min	Reduced aggregation during processing allowed a better protein degradation by pepsin	[115]
Fish protein hydrolysates	Glycation with ribose at 121 °C for 30, 60, and 90 min	Histamine release using RBL-2H3 cells		Histamine release in RBL-2H3 cells was reduced	Glycated fish protein hydrolysates reduced NO synthesis	[116]
Purified great snakehead parvalbumin	90 °C for 1, 2, 3 h	SDS-PAGE		The parvalbumin band intensity decreased as a function of heating time but was visible after 3 h heating	Parvalbumin maintained its typical structural properties after experiencing extensive thermal stroke	[117]
Purified sardine parvalbumin	70, 80, and 90 °C for 30, 60, and 120 min	Indirect non-competitive ELISA and dot blot	Rabbit anti-parvalbumin antibody	IgG binding to parvalbumin diminished 65% after 90 °C heating for 30 min	Heating was responsible for the reduction of antibody binding to purified sardine parvalbumin	[93]
			Human IgE	90% of patients showed reduced IgE binding, while 10% patients showed increased IgE binding		
Monkfish, Atlantic salmon, trout, pink ling, jewfish, pumpkin head trevally, swordfish, northern sand flathead, red gurnard, tiger flathead, and mosaic leatherjacket	100 °C for 45 min	Western blot	Anti-carp mAb Anti-cod mAb	Reduced IgG binding		[118]
Pilchard, cod, dory, bright redfish, sea mullet, pink ling, barramundi, blue threadfin, cobia, crimson snapper, flame snapper, grunter bream, jewfish, pink snapper, pumpkin head trevally, sweetlip emperor, saddletail snapper, striped snapper, yellowfin bream, yellowtail scad, northern sand flathead, and red gurnard	100 °C for 45 min	Western blot	Anti-carp mAb Anti-cod mAb	Consistent IgG binding as the raw protein extracts		
Coral trout, eastern school whiting, grass emperor, sand whiting, Spanish mackerel, yellowfin tuna, and tiger flathead	100 °C for 45 min	Western blot	Anti-carp mAb Anti-cod mAb	Increased IgG binding		
Purified sardine parvalbumin	90 °C for 1 h then pepsin digested for 30, 60, 120 min at pH 2, 37 °C	Indirect non-competitive ELISA and dot blot	Rabbit anti-parvalbumin antibody	Decreased IgG binding	Pepsin hydrolysis decreased the binding of IgG	
			Human IgE	All IgE-binding capacity was eliminated completely		
Whiting protein extracts	Soak in vinegar for 30 min and then heat at 100 °C for 5 min	Indirect non-competitive ELISA and Western blot	Anti-fish tropomyosin mAb	IgG-binding capacity decreased significantly regardless of different types of vinegar	Acidic pH changed the immunoreactivity and detectability of whiting	[119]
Whiting, cod, and red grouper protein extracts	Soak in vinegar for different periods (<1 min, 15 min, 30 min, and 60 min) and then heat at 100 °C for 5 min	Indirect non-competitive ELISA and Western blot	Anti-fish tropomyosin mAb	Whiting: IgG immunoreactivity decreased significantly after 15 min treatment; cod and grouper: IgG immunoreactivity decreased significantly even within 1 min treatment	Acid pH either altered tropomyosin conformation or lowered its solubility	
Whiting, cod, and red grouper protein extracts	100 °C for 5, 15, 30, and 60 min	Western blot	Human IgE	Prolonged vinegar cooking time significantly reduced the IgE immunoreactivity	Acid pH-induced protein denaturation	
Cod protein extracts	In vitro digestion at pH 1.25–5	Western blot	Human IgE	When pH ≤ 2.5, all proteins lost IgE-binding capability within 1 min; when 2.5 < pH ≤ 5, IgE immunoreactivity was still observed after 1 h digestion	Gastric pH could digest and degrade cod proteins. Those patients with abnormal gastric pH may be exposed to an increased allergenicity	[120]
		RAST inhibition	Human IgE	Digested cod proteins inhibited IgE binding as a function of time		
		Histamine release assay		Histamine release was only observed at high concentration of digests		
Whiff protein extracts	100 °C for 10 min	Western blot	Human IgE	More IgE-reactive bands were observed		[88]
Whiff protein extracts	100 °C for 10 min and then in vitro gastric digestion	Western blot	Human IgE	IgE bound to fragmented proteins even after 120 min; IgE binding to 24 kDa, 34 kDa, and 130 kDa proteins was weakened	Heating-induced protein degradation	
Purified whiff parvalbumin	100 °C for 10 min and then in vitro gastric digestion	Western blot	Human IgE	Immunoreactive parvalbumin monomer disappeared after 5 s digestion while its dimer was visible after 120 min	Heating generated dimers that were partially stable towards gastric digestion	
Purified Alaska pollock parvalbumin	Glycation with glucose, fructose, ribose, lactose, and galactose at 60 °C, 65% for 1 h	Indirect competitive ELISA	Rabbit antisera	Glycation with glucose and fructose enhanced both IgG and IgE binding, while glycation with ribose, lactose, and galactose decreased both IgG and IgE binding	Glycation changed protein conformation, which affected the specific recognition of antigen and antibody	[121]
			Human IgE			
Glass carp purified parvalbumin	Glycation with maltose	Indirect competitive ELISA	Rabbit anti-PV sera	Reduced IgG binding	Heat treatment was the major cause for decreased immunoreactivity	[36]
			Human IgE	Suppressed IgE binding	Heat treatment and Maillard reaction led to the structural change of parvalbumin	
Recombinant silver cap parvalbumin	Glycation with glucose at 60 °C for 72 h	Dot blot	Human IgE	Decreased IgE binding	Glycation sites were partially located at IgE-binding epitopes	[122]
		Rat basophilic leukemia assay		Reduced histamine release and secretion of IL-4 and TNF-α.		
Tuna	Canning	Double-blind placebo-controlled food challenge		All patients did not show sensitization and adverse reaction after consumption	Canning led to the formation of a homogenous mixture of different molecular weight fragments	[123]
		Immunoblot and indirect competitive ELISA	Human IgE	All sera showed minimal to absent IgE binding		
Salmon	Canning	Double-blind placebo-controlled food challenge		All patients did not show an adverse reaction after consumption	Canning led to a remarkable loss of definable protein bands on SDS-PAGE	
		Immunoblot and indirect competitive ELISA	Human IgE	Minimal IgE binding		
Haddock and rainbow trout	Hot smoking at 80–100 °C	Indirect competitive ELISA	Human IgE	83.3% of patients showed increased IgE binding	Novel bands at around 65 kDa were observed on SDS-PAGE	[38]
Tuna	Canning at high temperature (116–121 °C) and pressure for up to 14 h	Indirect competitive ELISA	Human IgE	All patients showed decreased IgE binding	No parvalbumin band was visible on the SDS-PAGE	
Atlantic cod	Drying	Indirect competitive ELISA	Human IgE	All patients showed increased IgE binding	Several novel bands from 70 to > 188 kDa were observed on SDS-PAGE	[38]
Atlantic cod	Dried cod soaked in a pH 11–12 lye solution and subsequently in cold water	Indirect competitive ELISA	Human IgE	All patients showed reduced IgE binding	Parvalbumin band intensity on SDS-PAGE was reduced 48%	
Atlantic cod	Cod dried after salting	Indirect competitive ELISA	Human IgE	58.3% of patients showed decreased IgE binding, while 33.3% patients showed increased IgE binding	Several novel bands from 70 to > 188 kDa were observed on SDS-PAGE	
Atlantic salmon	Cured in a mixture of sugar, spices, and salt	Indirect competitive ELISA	Human IgE	80% of patients showed reduced IgE binding, while 20% of patients showed 65 times more IgE binding	Parvalbumin band intensity on SDS-PAGE was reduced by 34%	
Atlantic salmon	Cold smoking at 20–30 °C after being cured for a day	Indirect competitive ELISA	Human IgE	80% of patients showed increased IgE binding	Novel bands at around 30 kDa were observed on SDS-PAGE	
Rainbow trout	Salted trout undergoes controlled enzymatic fermentation	Indirect competitive ELISA	Human IgE	81.8% of patients showed decreased IgE binding, while 18.2% patients showed 30 times more IgE binding	Parvalbumin band intensity on SDS-PAGE was reduced by 40%	
Herring	Pickled herrings are prepared in an acetic acid–salt brine	Indirect competitive ELISA	Human IgE	87.5% of patients showed decreased IgE binding	Few bands < 62 kDa were observed, and parvalbumin band intensity decreased on SDS-PAGE	
Salmon	Hydrolysis	Indirect competitive ELISA	Human IgE	Three patients showed more IgE binding	Absence of discernible bands and weak bands up to around 50 kDa	
Blue whiting	Hydrolysis	Indirect competitive ELISA	Human IgE	Two patients showed decreased IgE binding, while one patient showed more IgE binding	Absence of discernible bands and weak bands up to around 50 kDa	
Carp, catfish, chub mackerel, sardine, chinook salmon, albacore tuna, and mahi-mahi	Stored at −20 °C	Indirect non-competitive ELISA	IgG	A decrease in parvalbumin immunoreactivity was observed after 112-day storage, but parvalbumin was still considered stable at frozen stages	Less freeze-induced protein denaturation was observed in intact muscle. Frozen storage mainly altered myofibrillar proteins instead of sarcoplasmic proteins	[124]
Food-grade cod gelatin		Histamine release assay		10% of patients showed histamine release		
		Skin prick test		23.3% of patients showed positive results		
		Double-blind placebo-controlled food challenge		None of the patients showed allergic symptoms to 3.61 g fish gelatin		[125]
Yellowfin tuna gelatin		Western blot	Human IgE	3% of patients showed IgE binding	The manufacturing process eliminated the fish allergens	[32]
		Double-blind placebo-controlled food challenge		None of the patients showed allergic symptoms to 5 g fish gelatin		

Second, processing may lead to a decreased, unchanged, or even increased antigenicity and allergenicity. For example, the IgG immunoreactivity decreased when fish was processed into surimi [114]. Oral food challenge results showed that none of the 30 fish allergic patients developed allergic symptoms after the ingestion of fish gelatin [125]. This is because different food processing techniques cause (1) breaking of linear epitopes into small fragments; (2) changing its conformation to destroy conformational epitopes; (3) changing its conformation or exposure of the neoepitopes; and (4) masking of epitopes due to molecule attachment. Generally, linear epitopes are considered more stable than conformation epitopes. Heating, such as boiling, canning, and frying, might change protein solubility due to denaturation and protein aggregation as a function of temperature and time. As Kuehn et al. [126] reported, parvalbumin content in commercially thermally processed (smoked and canned) and laboratory-cooked (100 °C/10 or 20 min) fish decreased up to 60% and 25%, respectively, compared to unheated fish. Additionally, Wang [119] found a significant reduction of soluble protein concentration after treating whiting with vinegar. Hou [114] noticed a decrease in protein concentration after processing fish into surimi. Hydrolysis could reduce allergenicity/antigenicity but could also expose preexisting epitopes or create neoepitopes [127]. For example, trypsin hydrolysis generated two polypeptide fragments from cod that are allergenic [128]. Glycation, as another food processing method, could also increase or protein allergenicity [129]. After glycation, the digestibility and allergenicity of carp parvalbumin increased and decreased, respectively, due to glucose attachment to IgE epitopes [122]. The effect of glycation on parvalbumin allergenicity is also dependent on sugar structure, protein concentration, and glycation condition [121]. Non-thermal processing such as high hydrostatic pressure (HHP) could alter the secondary and tertiary structure of parvalbumin, which could effectively reduce its antigenicity [130]. It should be noted that a decrease in allergenicity in some fish allergic patients does not guarantee this function in other patients. Many studies reported different reactions to the same processed fish from different patients [32,38,93,124].

Third, current research on the effect of food processing on fish antigenicity and allergenicity has some limitations. Most of the studies used in vitro methods, such as Western blots and ELISAs, to study the effect of food processing on fish antigenicity and allergenicity. When using food allergic patient sera to evaluate IgE-binding capacity, it is important to consider the usage of pooled or individual patient sera. Pooled sera could rule out inter-individual differences, but they only reflect an average IgE reactivity [89]. When individual sera are used, the number of individuals should be taken into consideration. According to the WHO/IUIS, at least five sera of patients allergic to the respective allergen source should be used in allergenicity tests [46]. Those in vitro tests could not truly represent the fish allergenicity after human consumption. Few studies have applied skin prick tests, which may give false positive/negative results due to the different exposure routes. As for the ex vivo basophil activation test (BAT), the detection sensitivity also decreases over time [131]. In addition, recent studies have primarily focused on the major fish allergen, i.e., parvalbumin. Other major fish allergens, such as beta enolase, creatine kinase, and collagen, have not been fully studied. It is possible to generate new allergens from processing-induced protein–protein interaction. Some researchers have recently pointed out the urgency and necessity of further characterizing other fish allergens. For example, the first case of anaphylaxis due to the ingestion of gummy candy containing fish collagen was reported [132,133]. Kalic et al. [26] further proposed the relevance of investigating fish collagen. Additionally, Ruethers et al. [25] reported that fish tropomyosin, as a novel major fish allergen, is underestimated at the current stage.

## 5. Conclusions

Due to the fact that the production and consumption of fish have been increasing in recent years, the prevalence of fish allergy among different regions is also increasing. Currently, there is no cure for fish allergy, which can only be managed by strict avoidance of fish in the diet. The effect of food processing on fish proteins’ antigenicity and allergenicity is summarized in this concise review article. It is found that processing could alter a fish protein’s solubility and conformation and lead to an enhanced, impaired, or unchanged antigenicity and allergenicity. There are some limitations in recent studies. First, terminologies such as immunogenicity, antigenicity, and allergenicity are used interchangeably by different researchers. Second, due to the various fish species, recent research has mainly focused on the commonly consumed species. Moreover, among the 12 WHO/IUIS recognized fish allergens, parvalbumin is studied the most, whose antigenicity and allergenicity are mainly dependent on the existence of calcium. As for other fish allergens, although their antigenicity and allergenicity have been reported from different fish species, the characterization is not comprehensive. Third, current antigenicity/allergenicity evaluation methods are mainly conducted in vitro, which may not reflect the real immune response in reality. Future research can be conducted on (1) the development of official methods for evaluating proteins antigenicity, allergenicity, and immunogenicity; (2) the evaluation of other major fish allergens such as tropomyosin and collagen; (3) the investigation of food processing of less commonly consumed fish species.

## Figures and Tables

**Table 2:** Reported IgE epitopes of fish allergens from publications.

Species	Protein	UniProtKB Accession Number	Method	Amino Acid	Reported IgE Epitope	Comment	Reference
*Gadus morhua* (Baltic cod)	Parvalbumin beta	P02622	Epitope mapping	33–44	VGLDAFSADELK	Located on the junction between AB and CD domains Located on the junction between CD and EF domains Located on the calcium-binding loop of EF domain	[20]
				49–64	IADEDKEGFIEEDELK		
				65–74	LFLIAFAADL		
				88–96	AGDSDGDGK		
			Generation of mimotopes using phage display to mimic epitopes	23	S	The IgE binding epitopes are partially in accordance with previously defined peptides The identified IgE binding epitopes are conformational	[19]
				25–29	NHKAF		
				33–37	VGLTS		
				77–79	LTG		
				87	K		
				89–92	GDSD		
				94	D		
*Gadus morhua* (Atlantic cod)	Parvalbumin beta	Q90YK9	Epitope mapping Indirect non-competitive ELISA	95–109	GDGKIGVDEFGAMIKA	Corresponding to EF domain	[21]
	Parvalbumin beta	D3GME4	Indirect non-competitive ELISA	21-40	AGSFDHKKFFKACGLSGKST	It is a specific IgE epitope of Sco j 1	[22]
*Salmo salar* (Atlantic salmon)	Parvalbumin beta 2	Q91483	Peptide-based microarray immunoassay			No IgE epitopes were found	[75]
	Parvalbumin beta 1	Q91482	Peptide-based microarray immunoassay	1–18	MACAHLCKEADIKTALEA	Located in the AB domain Located in the AB domain; also reported in Baltic cod Located between CD and EF domains; also reported in Baltic cod	
				28–45	KTFFHTIGFASKSADDVK		
				61–85	VEELKLFLQNFCPKARELTDA		
Asian seabass	Parvalbumin beta 1	Q5IRB2	Indirect non-competitive ELISA	17–25	AACQAADSF	Both IgE binding regions are very similar to the identified regions from cod and carp	[24]
				106–109	LVKV		
*Salmo salar* (Atlantic salmon)	Tropomyosin alpha-1 chain	Q91472	Epitope mapping	43–57	LVALQKKLKGTEDEL	Both peptides were found in flathead gray mullet and Mozambique tilapia	[76]
				235–252	AETRAEFAERSVAKLEKT		

**Table 3:** Effect of calcium ion on parvalbumin antigenicity.

Sample	Method	Chelator in Blocker	Antibody	Chelator in Antibody Buffer	Major Result	Reference
Frog muscle protein extracts	Western blot	No	Human sera	5 mM EGTA	A decrease in IgE binding	[56]
		No	mAb PARV19	5 mM EGTA	No IgG binding	
Pacific mackerel protein extracts	Western blot	No	Rabbit anti-Pacific mackerel parvalbumin antiserum	5 mM EDTA	Same IgG binding	[30]
		No	mAb PARV19	5 mM EGTA	No IgG binding	
Scamp, sunfish, ocean perch, mullet, striped bass, catfish, pompano, red grouper, cobia, sheephead, tilapia, red snapper, basa, tra, amberjack, wahoo, Alaskan halibut, and yellowfin tuna protein extracts in coating buffer containing 10 mM EGTA	Indirect non-competitive ELISA	No	mAb PARV19	No	An increase in IgG binding	[79]
		No	mAb3E1	No	An increase in IgG binding	
Salmon and mullet protein extracts in water	Western blot	10 mM EDTA	mAb PARV19	10 mM EDTA	No IgG binding	[23]
		10 mM EDTA	mAb3E1	10 mM EDTA	IgG binding was not affected	
Salmon and mullet protein extracts in 5 mM EDTA in water	Western blot	No	mAb PARV19	No	IgG binding was enhanced	
		No	mAb3E1	No	IgG binding was enhanced	
Pacific mackerel parvalbumin	Indirect non-competitive ELISA	Unknown	Human sera	5 mM EGTA	Reduced IgE binding for 100% of patients	[80]
Cod, tuna, carp, salmon, and eel protein extracts	Western blot	No	Human sera	5 mM EGTA	More than 50% IgE binding reduction was observed in 64.2% of patients	[81]
Carp parvalbumin	Western blot	Unknown	Human sera	5 mM EGTA	100% of patients showed IgE binding reduction to a different extent	[77]
		Unknown	Anti-parvalbumin mAb	5 mM EGTA	18% IgG binding reduction	
Recombinant carp parvalbumin	Western blot	Unknown	Human sera	5 mM EGTA	100% of patients showed IgE binding reduction to a different extent	[82]

## Data Availability

Not applicable.
